# 

**Published:** 1901-11

**Authors:** 


### BOOKS RECEIVED.

Dose-Book and Manual of Prescription-Writing: with a List of Official Drugs and Preparations, and the more important Newer Remedies. By E. Q. Thornton, M. D., Demonstrator of Therapeutics, Jefferson Medical College, Philadelphia. Second Edition, Revised and Enlarged. Octavo, 362 pages, illustrated. Philadelphia and London: W. B. Saunders & Company, 1901. [Bound in flexible leather, $2.00 net.]

The Principles of Hygiene: A Practical Manual for Students, Physicians and Health Officers. By D. H. Bergey, A. M., M. D., First Assistant, Laboratory of Hygiene. University of Pennsylvania. Octavo volume of 495 pages, illustrated. Philadelphia and London: W. B. Saunders & Company, 1091. [Cloth, $3.00 net.

A Text-Book of Obstetrics. By Barton Cooke Hirst, M. D., Professor of Obstetrics in the University of Pennsylvania. Third edition, thoroughly revised and enlarged. Royal octavo, 873 pages, with 704 illustrations, many of them in colors. Philadelphia and London: W. B. Saunders & Company, 1901. [Cloth, $5.00 net.]

Pathological Technique. A Practical Manual for Workers in Pathological Histology, including Directions for the Performance of Autopsies and for Clinical Diagnosis by Laboratory Methods. By Frank P. Mallory, A. M., M. D., Associate Professor of Pathology, Harvard University Medical School; and James H. Wright, A. M., M. D., Instructor in Pathology, Harvard University Medical School. Second edition, revised and enlarged. Octavo, 432 pages, with 137 illustrations. Philadelphia and London: W. B. Saunders & Company, 1901. [Cloth, $3 00 net.]

Human Physiology. Prepared with special reference to Students of Medicine. By Joseph Howard Raymond, A. M., M. D., Professor of Physiology and Hygiene in the Long Island College Hospital, and Director of Physiology in Hoagland Laboratory, New York City. Second edition, entirely rewritten and greatly enlarged. Handsome octavo volume of 668 pages, 443 illustrations, 12 of them in colors, and 4 full-page lithographic plates. Philadelphia and London: W. B. Saunders & Company, 1901. [Cloth, $3.50 net.]

A Laboratory Course in Bacteriology. For the use of Medical, Agricultural, and Industrial Students. By Frederic P. Gorham, A. M., Professor of Biology, Brown University; Bacteriologist to the Health Department, Providence, R. I. 12mo volume of 198 pages, with 97 illustrations. Philadelphia and London: W. B. Saunders & Co., 1901. [Cloth, $1.25 net.]

Atlas and Epitomé of Special Pathologic Histology. By Docent Dr. Herman Bürck, of the Pathologic Institute of Munich. Edited by Ludvig Hektoen, M. D, Professor of Pathology in Rush Medical College, Chicago. Vol. II.—Liver; Urinary Organs: Sexual Organs; Nervous System: Skin; Muscles; Bones. With 123 colored illustrations on 60 lithographic plates and 192 pages of text. Philadelphia and London: W. B. Saunders & Company, 1901. [Cloth, $3.00 net.]

Modern Obstetrics: General and Operative. W. A. Newman, Dorland, A. M., M. D., Assistant Demonstrator of Obstetrics, University of Pennsylvania; Associate in Gynecology, Philadelphia Polyclinic. Second edition, rewritten and greatly enlarged. Handsome octavo, 797 pages, with 201 illustrations. Philadelphia and London: W. B. Saunders & Company, 1901. [Cloth, $4.00 net.]

A Text-Book of Diseases of Women. By Charles B. Penrose, M. D., Ph. D., formerly professor of Gynecology in the University of Pennsylvania. Fourth edition, revised. Octavo volume of 439 pages, handsomely illustrated. Philadelphia and London: W. B. Saunders & Company, 1901. [Cloth, $3.75 net.]

A Manual of the Practice of Medicine. By George Roe Lockwood, M. D., Professor of Practice in the Woman's Medical College of the New York Infirmary. Second edition, revised and enlarged. Octavo volume of 847 pages, with 79 illustrations and 20 full page plates. Philadelphia and London: W. B. Saunders & Company, 1901. [Cloth, $4.00 net.]

Diseases of the Digestive Organs in Infancy and Childhood. With Chapters on the Diet and General Management of Children, and Massage in Pediatrics. By Louis Starr, M. D., late Clinical Professor of Diseases of Children in the Hospital of the University of Pennsylvania; Consulting Pediatrist to the Maternity Hospital, Philadelphia, etc. Third edition, rewritten and enlarged. Illustrated. P. Blakiston's Son & Co., Philadelphia, 1901. [Price, $3.00 net.]

The Principles and Practice of Medicine. Designed for the use of Practitioners and Students. By Wm. Osler, M. D., Fellow of the Royal Society; Fellow of the Royal College of Physicians, London; Professor of Medicine in the John Hopkins University and Physician-in-Chief to the John Hopkins Hospital, Baltimore, etc. Octavo, pp. 1200 Fourth edition. New York: D. Appleton & Company, 1901. [Price, cloth, $5.50; sheep, $6-50; half-morocco, $7.00]

Pediatrics. The Hygienic and Medical Treatment of Children. By Thomas Morgan Rotch, M. D., Professor of the Diseases of Children, Harvard University. Third edition, rearranged and rewritten. Octavo pp. 1042. Illustrated by numerous engravings in the text and by colored plates. Philadelphia and London: J. B. Lippincott Company, 1901.

A Practical Treatise on Diseases of the Skin. By John V. Shoemaker, M. D., LL. D. Professor of Skin and Venereal Diseases in the Medico-Chirurgical College and Hospital, Philadelphia; Physician to the Philadelphia Hospital for Diseases of the Skin, etc. Octavo, pp. 905. Fourth edition, revised and enlarged, New York: D. Appleton & Company. 1901. [Price, cloth, $5.00; sheep, $6.00.]

A Manual of Bacteriology By Herbert U. Williams, M. D., Professor of Pathology and Bacteriology in the Medical Department of the University of Buffalo. With ninety illustrations. Second edition, revised and enlarged. P. Blakiston's Son & Co., Philadelphia, 1901. [Price, $1.50 net.

The Technique of Surgical Gynecology. Devoted exclusively to a description of the technique of gynecological operations By Austin H Goelet, M. D., Professor of Gynecology in the New York School of Clinical Medicine. Duodecimo, pp. 340. Illustrated. New York: International Journal of Surgery Co. 1901.

Warwick of the Knobs. A story of Stringtown County, Kentucky. By John Uri Lloyd, Author of "Stringtown on the Pike," etc. With photographic illustrations of Knob County. New York: Dodd, Mead & Company. 1901. [Price, $1.50.]

Transactions of the Rhode Island Medical Society. Volume VI., Part II. George D. Hersey, Librarian. 1900.

The Physician's Pocket Account Book. Consisting of a manila-bound book of 208 pages and a leather case. By J. J. Taylor, M. D. Philadelphia: Published by The Medical Council, Twelfth and Walnut Streets. [Price, $1.00 complete; subsequent books to fill the case, 40 cents each, or three for $1.00.]

Transactions of the Medical Association of the State of Alabama (The State Board of Health). Annual meeting held at Selma, April 16–19, 1901. G. P. Waller, M. D., Secretary.

A Text-Book of Bacteriology. By George M. Sternberg, M. D., LL. D., Surgeon-General U. S. Army; Ex-President of the American Medical Association and of the American Public Health Association, etc. Octavo, pp. 718. Illustrated by heliotype and chromo-lithographic plates and 200 engravings. Second revised edition. New York: William Wood & Co. 1901. [Price, cloth, $4.50 net; brown sheep, $5.25 net.]

A Guide to Lectures and Laboratory Work for Beginners in Chemistry. Specially adapted for students of medicine, pharmacy and dentistry. By W. Simon, Ph. D., M. D., Professor of Chemistry in the College of Physicians and Surgeons of Baltimore, in the Maryland College of Pharmacy, and in the Baltimore College of Dental Surgery. Seventh edition, thoroughly revised and much enlarged. In one octavo volume of 613 pages, with 66 engravings, 1 colored spectra plate and 8 colored plates representing 64 of the most important chemical reactions. Philadelphia and New York: Lea Brothers & Co. 1901. [Price, cloth, $3.00 net.]

Progressive Medicine. Volume III., September, 1901. A quarterly digest of advances, discoveries and improvements in the medical and surgical sciences. Edited by Hobart Amory Hare, M. D., Professor of Therapeutics and Materia Medica in the Jefferson Medical College of Philadelphia Octavo, 428 pages, 16 illustrations. Philadelphia and New York: Lea Brothers & Co, [Per annum, in four cloth-bound volumes, $10.00.]

Report of the Commissioner of Education for the Year 1899-1900. Volume I. Washington: Government Printing Office. 1901.

Materia Medica, Pharmacy, Pharmacology and Therapeutics. By W. Hale White, M. D, F. R. C. P., Physician to and Lecturer on Medicine at Guy's Hospital, London; Author of A Text-Book of General Therapeutics. Edited by Reynold W. Wilcox, M. A., M. D., LL. D., Professor of Medicine and Therapeutics at the New York Post-Graduate Medical School and Attending Physician to the Hospital; Visiting Physician to St. Mark's Hospital; President of the American Therapeutic Society; Fellow of the American Academy of Medicine, etc. Fifth American edition, thoroughly revised. Philadelphia: P. Blakiston's Son & Co. 1901. [Price, $3.00 net.]

Twentieth Annual Report of the State Board of Health of New York. For the year ending December 31, 1899 Transmitted to the legislature February 15, 1900. Albany: James B. Lyon, State Printer. 1901.

Handbook on Sanitation. A manual of theoretical and practical sanitation. For students and physicians; for health, sanitary, tenement-house, plumbing, factory, food and other inspectors; as well as for candidates for all municipal sanitary positions. By George M. Price, M. D., Medical Sanitary Inspector, Department of Health, New York City; Inspector New York Sanitary Aid Society of the 10th Ward, 1885; Manager Model Tenement-houses of the New York Tenement-house Building Company, 1888; Inspector New York State Tenement-house Commission, 1895. Duodecimo, pp. 329. Illustrated. New York: John Wiley & Sons. 1901. [Price, $1.50 net.]

